# Impaired adenosine-mediated angiogenesis in preeclampsia: potential implications for fetal programming

**DOI:** 10.3389/fphar.2014.00134

**Published:** 2014-06-05

**Authors:** Carlos Escudero, James M. Roberts, Leslie Myatt, Igor Feoktistov

**Affiliations:** ^1^Vascular Physiology Laboratory, Group of Investigation in Tumor Angiogenesis, Group of Research and Innovation in Vascular Health, Department of Basic Sciences, Faculty of Sciences, Universidad del Bío-BíoChillán, Chile; ^2^Magee-Womens Research Institute, Department of Obstetrics, Gynecology, and Reproductive Sciences, Department of Epidemiology and Clinical and Translational Science Institute, University of PittsburghPittsburgh, PA, USA; ^3^Center for Pregnancy and Newborn Research, University of Texas Health Science CenterSan Antonio, TX, USA; ^4^Division of Cardiovascular Medicine, Department of Medicine, Vanderbilt UniversityNashville, TN, USA; ^5^Department of Pharmacology, School of Medicine, Vanderbilt UniversityNashville, TN, USA

**Keywords:** adenosine receptors, angiogenesis, placenta, preeclampsia, programming

## Abstract

Preeclampsia is a pregnancy-specific syndrome, defined by such clinical hallmarks as the onset of maternal hypertension and proteinuria after 20 weeks of gestation. The syndrome is also characterized by impaired blood flow through the utero-placental circulation and relative placental ischemia, which in turn, may generate feto-placental endothelial dysfunction. Endothelial dysfunction in offspring born from preeclamptic pregnancies has been associated with an increased risk of cardiovascular disease, including hypertension, later in life. Interestingly, diminished endothelial function, manifested by low angiogenic capacity, leads to hypertension in animal studies. Recently, we have shown that the adenosine receptor A_2A_/nitric oxide/vascular endothelial growth factor axis is reduced in human umbilical vein endothelial cells derived from preeclamptic pregnancies, an effect correlated with gestational age at onset of preeclampsia. We and others suggested that impaired vascular function might be associated with high cardiovascular risk in offspring exposed to pregnancy diseases. However, we are not aware of any studies that examine impaired adenosine-mediated angiogenesis as a possible link to hypertension in offspring born from preeclamptic pregnancies. In this review, we present evidence supporting the hypothesis that reduced adenosine-mediated angiogenesis during preeclamptic pregnancies might be associated with development of hypertension in the offspring.

## INTRODUCTION

Preeclampsia is a major cause of maternal and infant morbidity and mortality worldwide (Xiong et al., 2002; Duley, 2009). Stillbirth is more common in preeclamptic pregnancies, and one third of infants of preeclamptic women exhibit growth restriction. Furthermore, the appropriate management of preeclampsia (i.e., delivery of women with increasingly severe disease) is responsible for 8% of preterm births, with attendant increased morbidity and mortality (Sibai et al., 2005; Villar et al., 2006). In the last 30 years, it has become evident that the fetal intrauterine environment has long-lasting consequences for the infant. Low or high birth weight, prematurity, gestational diabetes, and hypertension not only have an impact on perinatal outcomes; they also have long-term consequences, increasing the risk of neurological disability, obesity, and cardiovascular disease in adult life (Gluckman and Hanson, 2004; Hanson and Gluckman, 2008; Krause et al., 2009). The multi-organ dysfunction syndrome associated with preeclampsia could, directly or indirectly, affect the intrauterine environment.

The underlying pathophysiology of preeclampsia includes dysregulation of endothelial function in both the maternal and the feto-placental circulation (Roberts and Escudero, 2012). Several groups have suggested that long-term complications in offspring from preeclamptic pregnancies might be associated with loss of the endothelium’s ability to regulate vascular tone by synthesizing vasoactive molecules. However, the endothelium is also responsible for the generation of new vessels through the process of angiogenesis. Imbalance of angiogenic factors (i.e., reduction in the activity of pro-angiogenic factors in association with high activity/expression of the anti-angiogenic factors) is a well-characterized feature of preeclampsia. The implication of this imbalance for the occurrence of long-term complications in offspring from preeclamptic pregnancies is not yet clear. Since adequate formation of blood vessels is required for controlling blood pressure and for tissue repair, it is likely that impaired angiogenesis may contribute to future cardiovascular risk in the newborn “exposed” to preeclampsia *in utero*.

Adenosine is a naturally occurring nucleoside, which is increased in the feto-placental circulation with preeclampsia (Yoneyama et al., 1996; Escudero et al., 2009; Espinoza et al., 2011). It plays an important role in controlling the production and action of pro-angiogenic factors such as vascular endothelial growth factor (VEGF), as well as anti-angiogenic factors including soluble fms-like tyrosine kinase-1 (sFlt-1; George et al., 2010) and, through this mechanism, may control placental angiogenesis (Escudero et al., 2012). Therefore, it is plausible that dysfunctional adenosine-mediated angiogenesis *in utero* and after birth may contribute to long-term complications in offspring from preeclamptic pregnancies.

## PATHOPHYSIOLOGY OF PREECLAMPSIA: CURRENT HYPOTHESES

Preeclampsia has been defined by the onset of hypertension and proteinuria after 20 weeks of gestation and is additionally characterized by maternal endothelial dysfunction (Roberts et al., 1989). However, recently The American College of Obstetricians and Gynecologists has stated that proteinuria is no longer absolutely required for diagnosis of preeclampsia (ACOG, Task Force of Hypertension in Pregnancy, 2013). Alternatively, diagnosis may be established by the presence of hypertension associated with thrombocytopenia (platelet count less than 100.000/μL), impaired liver functions (elevated blood concentrations of liver transaminases up to twice the normal concentration), development of renal insufficiency (serum creatinine concentration greater than 1.1 mg/dL or a doubling of the serum creatinine concentration in the absence of other renal diseases), pulmonary edema, or new-onset cerebral or visual disturbances.

Current thinking on the pathophysiology of preeclampsia suggests that impaired invasion of trophoblastic cells into the maternal vascular bed leads to aberrant transformation of uterine resistance vessels to large diameter capacitance vessels (Burton et al., 2009a,b). This reduces maternal blood flow to the placenta and generates relative ischemia. Failure to increase the terminal caliber of the spiral arteries results in increased velocity of blood entering the intervillus space, with consequent shear stress on the fetal villus trophoblast (Burton et al., 2009b), leading to cell damage, detachment, and the release of cell fragments into the maternal circulation (Tannetta et al., 2013). These fragments contain elements that may impair maternal endothelial function and generate a vicious cycle that will chronically affect maternal and feto-placental endothelial function.

Among molecules released from the placenta, sFlt-1 has become a focus of study in preeclampsia. It is increased in the blood of women prior to and during clinical preeclampsia and has the potential to blunt angiogenic responses. Importantly, reduction of the plasma level of sFlt-1 in women with preterm preeclampsia using dextran sulfate apheresis has been reported to reduce proteinuria and blood pressure and prolong pregnancy by 23 days without apparent adverse events for mother or fetus (Thadhani et al., 2011). Moreover, the injection of mice with adenovirus expressing sFlt-1 results in pathophysiological findings resembling preeclampsia (Maynard et al., 2003; Bytautiene et al., 2011; Murphy et al., 2012). This animal model has been employed to study not only the pathophysiology of preeclampsia but also vascular alterations in the offspring (Lu et al., 2007a; Byers et al., 2009; Bytautiene et al., 2011).

## DEVELOPMENTAL ORIGINS OF ADULT DISEASE AFTER PREECLAMPSIA

The fetal programming hypothesis proposes that chronic diseases may originate through adaptations of the fetus to an adverse intrauterine environment. These adaptations may include changes in the vascular, metabolic, or endocrine systems. Those changes permanently affect function in adult life.

Numerous epidemiological studies suggest an important role for the adverse intrauterine environment in the development of schizophrenia, depression, cardiovascular diseases, stroke, diabetes, cancer, pulmonary hypertension, osteoporosis, polycystic ovarian syndrome, and other conditions in adult life. These observational relationships are supported by animal experiments in which effects on fetal growth via manipulation of maternal nutrition or reduction of blood flow to the placenta (by various approaches) result in obesity, increased blood pressure, and other cardiovascular abnormalities in the offspring later in life (Hanson and Gluckman, 2008; Glover, 2011; Davis et al., 2012b). Applying this concept to preeclampsia brings in a number of other insults which may trigger programming. These include increased oxidative stress and elevated concentration of anti-angiogenic factors, which can also result in growth restriction or premature deliveries.

### PREECLAMPSIA AND LONG-TERM ADVERSE OUTCOMES IN THE OFFSPRING

Many epidemiological studies (Kajantie et al., 2009; Wu et al., 2009, 2011; Davis et al., 2012a,b; Lawlor et al., 2012) indicate that preeclampsia is associated with long-term adverse outcomes in the offspring. The majority of studies (Palti and Rothschild, 1989; Seidman et al., 1991; Tenhola et al., 2003, 2006; Vatten et al., 2003; Swarup et al., 2005; Hiller et al., 2007; Oglaend et al., 2009; Kvehaugen et al., 2010; Lazdam et al., 2010; Palmsten et al., 2010; Lawlor et al., 2012), but not all (Ounsted et al., 1983; Jayet et al., 2010; Belfort et al., 2012; Lawlor et al., 2012) report that children and adolescents who were exposed to preeclampsia or hypertension in pregnancy exhibit higher systolic and diastolic blood pressure compared with non-exposed children or adolescents. These studies were reviewed in a recent meta-analysis (Davis et al., 2012a), which included individuals aged 4–30 years, born at term from preeclamptic pregnancies. This meta-analysis concluded that offspring born from preeclamptic women had ~2 mm Hg greater systolic and ~1.3 mm Hg greater diastolic blood pressure than individuals born from normotensive pregnancies. Interestingly, according to their prediction, “if the 2.4 mmHg difference in systolic blood pressure tracks into adulthood (Chen and Wang, 2008), this difference would be associated with an ~8% increased risk of mortality from ischemic heart disease and 12% increased risk from stroke” (Davis et al., 2012a). Based on a study in a large population of preeclamptic pregnancies, Kajantie et al. (2009) reported that the risk for stroke in subjects born from preeclamptic pregnancies was twice that of controls born from normotensive pregnancies. Other studies have described an increased risk for pulmonary hypertension (Jayet et al., 2010), metabolic and endocrine disease (Wu et al., 2009, 2011), depression (Tuovinen et al., 2010), cerebral palsy (Szymonowicz and Yu, 1987), poor cognitive outcome (Cheng et al., 2004), or intellectual disabilities (Griffith et al., 2011) in children born of preeclamptic pregnancies compared to non-exposed children.

These clinical and epidemiological observations are supported by a recent review of animal models of preeclampsia (Davis et al., 2012b), including those generated by systemic hypoxia, by mechanical reduction of maternal uterine artery blood flow, in genetically modified animals lacking endothelial nitric oxide synthase (eNOS), or by overexpression of sFlt-1 by infection with adenovirus carrying this protein. Notwithstanding differences in design and outcome of these models, the conclusion was that “animal studies support the potential relevance of these insults to programming of offspring blood pressure.”

Although fetal programming by preeclampsia is suggested by human and animal studies, it is not easy to determine whether preeclampsia *per se* leads to high cardiovascular risk in the offspring or whether related factors, such as intrauterine growth restriction or preterm delivery, contribute. To allay these concerns, most studies have excluded individuals exposed to preterm delivery or intrauterine growth restriction associated with preeclampsia. Interestingly, the risks for hypertension, impaired neurological function, and stroke in offspring from preeclamptic pregnancies remain significant (Kajantie et al., 2009; Tuovinen et al., 2010, 2012). Moreover, a study performed in brothers exposed, or not, to preeclampsia suggested that impaired vessel function was associated with preeclampsia *per se* rather than genetic predisposition (Jayet et al., 2010). It is plausible, then, that exposure to preeclampsia *in utero* can predispose to adverse outcomes later in life.

Understanding the underlying mechanisms might suggest interventions to prevent the occurrence of future chronic disease in offspring exposed to preeclampsia. Several groups (Jayet et al., 2010; Lazdam et al., 2010; Kvehaugen et al., 2011; Davis et al., 2012a,b; Lawlor et al., 2012), including ours (Escudero and Sobrevia, 2008; Escudero et al., 2012), have presented evidence of endothelial dysfunction in the feto-placental circulation in preeclampsia, which may be a precursor to the long-term complications observed in offspring born of preeclamptic pregnancies.

## ENDOTHELIAL DYSFUNCTION AND IMPAIRED ANGIOGENESIS IN OFFSPRING BORN FROM PREECLAMPTIC PREGNANCIES

Endothelial dysfunction is a pathological state characterized by an imbalance between vasodilators and vasoconstrictors produced by (or acting on) the endothelium (Brunner et al., 2005). Infants born of preeclamptic pregnancies have evidence of endothelial dysfunction shortly after delivery and months to years later (Davis et al., 2012a,b; Sobrevia et al., 2012; Wadsack et al., 2012). For instance, reduced flow-mediated vasodilatation (a hallmark of endothelial dysfunction) has been reported in children born from preeclamptic pregnancies as compared to children born from normotensive pregnancies (Jayet et al., 2010; Lazdam et al., 2010; Kvehaugen et al., 2011; Davis et al., 2012b). In animal studies using mice, male offspring born from mothers with preeclamptic-like syndrome, generated by administration of adenovirus carrying sFlt-1, exhibited high blood pressure (Lu et al., 2007a,b) and increased vascular reactivity (Byers et al., 2009; Bytautiene et al., 2011) compared to controls.

It is also clear that endothelial cells are main contributors to angiogenesis (Shibuya, 2006; Escudero et al., 2009), which leads to the growth of new blood vessels from pre-existing ones. The endothelium participates in angiogenesis through several processes, which include cell proliferation/migration, tube formation, as well as synthesis and release of pro-angiogenic factors including VEGF (Shibuya, 2013). In addition, neovasculogenesis, a process of blood vessel formation occurring by *de novo* production of endothelial cells, can occur not only at the embryonic stage but also in adult life (Risau, 1997). Endothelial progenitor cells (EPCs) play a critical role in postnatal blood vessel formation and vascular homeostasis. In preeclampsia, impaired feto-placental angiogenesis (Escudero et al., 2009, 2012, 2014) and neovasculogenesis (due to the reduced number of EPCs found in umbilical cord blood (Kwon et al., 2007; Xia et al., 2007; Monga et al., 2012) may be a result of endothelial dysfunction.

Reduced placental levels of several pro-angiogenic factors have been reported in the feto-placental circulation in early-onset preeclampsia (EOPE) when these are compared to late-onset preeclampsia (LOPE) or to age-matched controls (Gellhaus et al., 2006; Junus et al., 2012). Microarray analysis reveals lower expression of at least two angiogenesis-associated transcripts (Egfl7 and Acvrl1) in EOPE compared to LOPE or age-matched controls (Junus et al., 2012). Recently, we have reported that the proliferation/migration of human umbilical vein endothelial cells (HUVEC) is reduced in EOPE compared to LOPE or controls, whereas cells from LOPE exhibit elevated proliferation/migration compared to controls (Escudero et al., 2012). These reports suggest that angiogenesis could be modified in the feto-placental circulation in preeclampsia.

**FIGURE 1:**
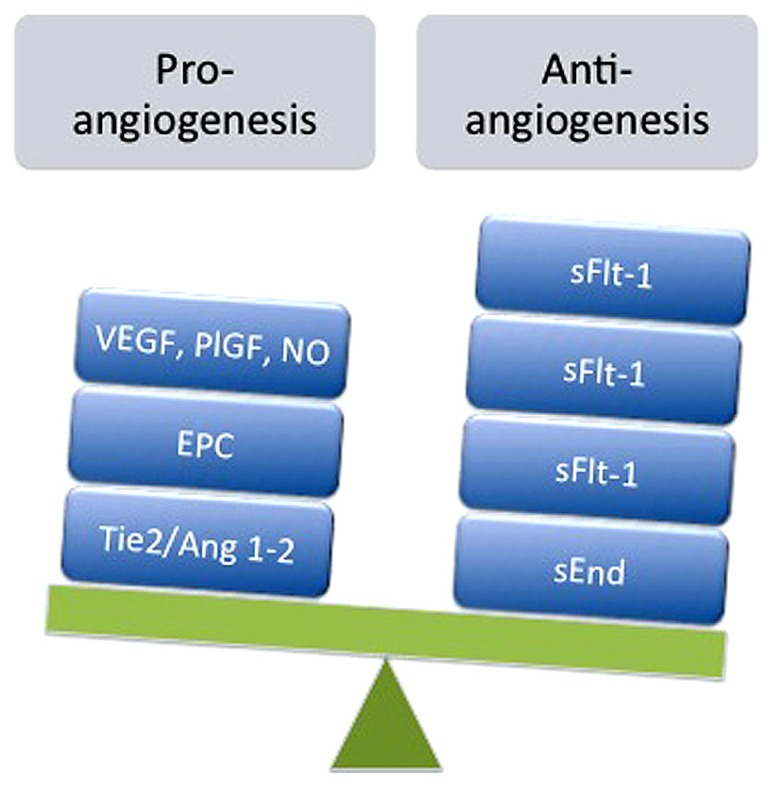
**Imbalance of pro- and anti-angiogenesis in feto-placental circulation during preeclampsia.** Represented is the reduction in expression and/or action of pro-angiogenic factors such as vascular endothelial growth factor (VEGF), placental growth factor (PlGF), adenosine, nitric oxide (NO), angiopoietin 1 (Ang1) and 2 (Ang2), and angiopoietin tyrosine kinase (Tie2), as well as reduction in the number of endothelial progenitor cells (EPC) exhibited in umbilical blood derived from preeclamptic pregnancies. On the other hand, elevated levels of anti-angiogenic factors such as soluble receptor type 1 of VEGF (sFlt-1) and soluble endoglin receptor (sEnd) have been also reported in umbilical circulation from preeclampsia.

The mechanisms underlying impaired neovascularization in feto-placental circulation during preeclampsia are under investigation and may be associated with the reduced numbers of EPCs observed in umbilical blood (Kwon et al., 2007; Xia et al., 2007; Monga et al., 2012), an increase in circulating anti-angiogenic factors such as sFlt-1 (Staff et al., 2005; Tsao et al., 2005) and soluble endoglin (sEnd; Staff et al., 2007), or reduced expression and activity of pro-angiogenic signals such as VEGF (Lyall et al., 1997; Andraweera et al., 2012; Kim et al., 2012) or adenosine (Escudero et al., 2012). As presented in **Figure 1**, this imbalance is manifested mainly by elevation of sFlt-1and sEnd, associated with reduced numbers of EPCs, which may nullify pro-angiogenic signals from VEGF and placental growth factor (PlGF).

Several prior studies have examined feto-placental tissue; however, few studies (see for instance Staff et al., 2005; Tsao et al., 2005; Kvehaugen et al., 2010, 2011) have assessed endothelial dysfunction postnatally in newborn infants or children exposed to preeclampsia. Considering that placental blood vessels on the fetal side form a continuous network with fetal systemic circulation, it is pertinent to ask whether offspring exposed to preeclampsia exhibit endothelial dysfunction and impaired angiogenesis after birth.

To the best of our knowledge, there is no direct answer to this question. However, indirect evidence includes increased concentrations of sFlt-1 in the fetus from preeclamptic pregnancies as measured in umbilical cord blood (Staff et al., 2005; Tsao et al., 2005). Offspring with high blood pressure whose parents also had high blood pressure showed fewer capillaries in the dorsum of the finger compared with either offspring with low blood pressure whose parents were either normotensive or hypertensive or hypertensive offspring whose parents were normotensive (Noon et al., 1997). More recently, Yu et al. (2012) reported that children born from preeclamptic pregnancies exhibited a 45% reduction in the risk of retinopathy of prematurity, a well-described example of pathological angiogenesis in premature infants, compared to preterm babies born from normotensive pregnancies. Moreover, Stark et al. (2009), studying blood flow immediately after birth in the microcirculation of children born from preeclamptic pregnancies, found altered fetal microvascular structure and function, particularly in male newborns.

In animal studies, at postnatal day 7, rat pups from spontaneously hypertensive mothers, exposed to hypoxic-ischemic brain injury, exhibited less brain damage than pups from normotensive mothers (Letourneur et al., 2012). Interestingly, this apparently protective phenomenon was associated with deficits in motor coordination and spatial learning in pups from hypertensive, compared to normotensive mothers. These results could be interpreted as a consequence of impaired angiogenesis. This could reduce the area of the lesion but also impair tissue recovery after ischemic insult in the brain. Therefore, it is plausible that offspring born from preeclamptic pregnancies may exhibit reduced angiogenic processes after birth, which may lead to cardiovascular complications later in life.

The latter concept is supported by the following findings: (1) VEGFR2 gene expression decreases with development (Greene et al., 2011). Also, vessel branching in the brain increases until 10 days postpartum and stabilizes to adult levels between days 10 and 25 in mice (Harb et al., 2013); (2) mature (4–5 month old) mouse brains lose their ability to undergo angiogenesis in response to hypoxia (Harb et al., 2013), suggesting that the process of angiogenesis, even in stressful conditions, is limited after birth; (3) VEGF production and activity are both impaired in the feto-placental circulation during preeclampsia (Lyall et al., 1997; Andraweera et al., 2012; Kim et al., 2012); (4) Inhibition of angiogenesis with humanized antibodies targeting VEGF or orally active small tyrosine kinase inhibitors targeting VEGF receptors is commonly associated with severe hypertension (Lankhorst et al., 2013); (5) Loss of microvessel growth has been reported to precede elevations in blood pressure (Murfee and Schmid-Schonbein, 2008); (6) Programming of elevated blood pressure in the offspring has been associated with a reduced angiogenic capacity of vessels cultured *in vitro* (Pladys et al., 2005). Taking all these data into account, we believe that abnormal angiogenic processes present after birth in offspring born from preeclamptic pregnancies may contribute to elevation in blood pressure later in life.

## OVERVIEW OF ADENOSINE RECEPTORS

### ADENOSINE RECEPTORS AND ANGIOGENESIS

Adenosine is a purinergic nucleoside which controls several physiological processes, including angiogenesis and vasculogenesis. Adenosine activates a family of G-coupled adenosine receptors, A_1_AR, A_2A_AR, A_2B_AR, and A_3_AR (Olah and Stiles, 2000; Jacobson and Gao, 2006). All of the adenosine receptors have been implicated in the modulation of angiogenesis (see Table 1). Briefly, stimulation of A_1_AR on embryonic EPCs promotes their adherence to the vascular endothelium, suggesting an important role for this receptor subtype in vasculogenesis (Ryzhov et al., 2008). A_1_AR have also been reported to upregulate VEGF production from monocytes, thus promoting angiogenesis (Clark et al., 2007).

**Table 1:** Summary of participation of adenosine receptor in angiogenesis using human cells.

AR	*K*_d_ (nM)	Angiogenic process	Cell type	Reference
A_1_	3–30	⇧ Migration	EPC	Ryzhov et al. (2008)

A_2A_	1–20	⇧ VEGF expression	Macrophages	Pinhal-Enfield et al. (2003), Ernens et al. (2010)
		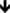 Thrombospondin 1 expression	HMVEC	Desai et al. (2005)
		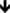 sFlt-1 release	Macrophages	Leonard et al. (2011)
		⇧ mFlt-1 expression	Macrophages	Leonard et al. (2011)
		⇧ Proliferation/migration and VEGF expression	HUVEC	Escudero et al. (2012)

A_2B_	5.000–20.000	⇧ Permeability	HUVEC-PMN	Lennon et al. (1998)
		⇧ VEGF expression	HMVEC	Ryzhov et al. (2014)
		⇧ Migration	HREC	Afzal et al. (2003)
		⇧ VEGF, IL-8 and bFGF expression	HMEC-1	Feoktistov et al. (2002)
		⇧ Migration	EPC	Rolland-Turner et al. (2013)
		⇧ VEGF and IL-8 expression	Foam cell	Gessi et al. (2010)
		⇧ IL-8 secretion	Melanoma cells, HT29	Merighi et al. (2007,2009)
		⇧ VEGF expression	HUVEC under hypoxia	Feoktistov et al. (2004)
		⇧ VEGF and IL-8 expression	HMEC-1	Feoktistov et al. (2003)
		⇧ Proliferation/migration and tube formation and VEGF expression	HREC	Grant et al. (1999,2001)
A_3_	> 1.000	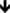 Migration and tube formation	HUVEC	Kim et al. (2013)
		⇧ VEGF and IL-8 expression	Foam cell	Gessi et al. (2010)
		⇧ VEGF and IL-8 expression	Melanoma cells	Merighi et al. (2009)
		⇧ VEGF expression	HT29	Merighi et al. (2007)
		⇧ Angiopoietin-2 expression	HMEC-1	Feoktistov et al. (2003)

*K_d_ (nM) for adenosine. VEGF, vascular endothelial growth factor; sFlt-1, soluble; mFlt-1, membrane-linked receptor type 1 for VEGF; IL-8, Interleukin 8; bFGF, basic fibroblast growth factor; HMVEC, human microvascular endothelial cells; HUVEC, human umbilical vein endothelial cells; PMN, polymorphonuclear leukocyte; HREC, human retinal endothelial cells; HMEC-1, human microvascular endothelial cell line 1; EPC, endothelial progenitor cells; HT-29, human colon adenocarcinoma. Increase (⇧) and decrease (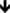) of pro or anti-angiogenic processes; AR, adenosine receptor.*

Depending on the tissue or cell studied, A_2A_AR and A_2B_AR can play a dominant role in the regulation of angiogenic factors. For example, A_2B_AR upregulates the pro-angiogenic factors VEGF, basic fibroblast growth factor (bFGF), insulin-like factor-1, and interleukin 8 (IL-8) in human microvascular endothelial cells (Grant et al., 1999; Feoktistov et al., 2002). Conversely, A_2A_AR is reported to upregulate VEGF in macrophages (Leibovich et al., 2002; Pinhal-Enfield et al., 2003). Stimulation of A_3_AR in mast cells and some tumors can result in upregulation of pro-angiogenic factors, complementing the actions of adenosine mediated via A_2B_AR (Feoktistov et al., 2003). Of interest, stimulation of A_2A_AR in HMEC-1 inhibits the release of the anti-angiogenic factor thrombospondin 1, providing yet another means by which adenosine may regulate angiogenesis (Desai et al., 2005; see Table 1).

While A_2A_AR and A_2B_AR have been shown to mediate the proliferative actions of adenosine in human retinal microvascular endothelial cells (Grant et al., 1999, 2001), HUVEC (Feoktistov et al., 2004; Escudero et al., 2012), or porcine coronary artery and rat aortic endothelial cells (Dubey et al., 2002), it remains unclear whether A_1_AR and A_3_AR are functionally expressed and what role, if any, they play in endothelial cells (Wyatt et al., 2002; Schaddelee et al., 2003).

### ADENOSINE RECEPTORS AND INTRACELLULAR PATHWAYS DURING ANGIOGENESIS

Although some data suggest that cAMP may play a role in the pro-angiogenic effects of adenosine in certain cells (Takagi et al., 1996), other studies show that upregulation of angiogenic factors is mediated via coupling to Gq, possibly involving mitogen-activated protein kinase (MAPK) pathways (Grant et al., 1999, 2001; Feoktistov et al., 2002; Ryzhov et al., 2014). Further studies, using HMEC-1 demonstrated that adenosine receptor-dependent upregulation of VEGF production was associated with an increase in VEGF transcription, activator protein 1 (AP-1) activity and transcription factor JunB (JunB) accumulation (Ryzhov et al., 2014).

Mechanistically, A_2B_AR which are coupled to both Gs and Gq proteins (Feoktistov and Biaggioni, 1995) increase JunB protein levels and VEGF production via stimulation of protein lipase C and extracellular signal-regulated kinase (ERK), which are possibly linked by the calcium diacylglycerol guanine nucleotide exchange factor (CalDAG-GEF)–Ras-proximate-1 (Rap1) pathway (Ryzhov et al., 2014). These effects were protein kinase A (PKA)-independent because the PKA inhibitors had no effect on the A_2B_AR-dependent increase in JunB protein levels and VEGF production. Because VEGF secretion and reporter promoter activity induced by the adenosine analog 5′-*N*-ethylcarboxamido-adenosine (NECA) were inhibited by the expression of a dominant, negative JunB or by JunB knockdown, these data suggest an important role for the A_2B_ receptor-dependent upregulation of JunB in VEGF production in various cell types, including endothelial cells (Ryzhov et al., 2014).

Another study, in HUVEC, reported that adenosine-mediated activation of ERK may involve an exchange protein activated by cAMP (Epac), a component of a family of cAMP-activated guanine nucleotide exchange factors for Rap GTPases (Fang and Olah, 2007). Thus, A_2B_AR, coupled to Gα_s_ promotes activation of adenylyl cyclase and an increase in intracellular cAMP. In turn, cAMP activates Epac 1, which may then activate a cascade of RapGTPase, B-Raf, and finally, ERK (Fang and Olah, 2007), demonstrating an alternative pathway for ERK activation involved in upregulation of pro-angiogenic proteins.

In addition to JunB, the mediators downstream of ERK and p38 MAPK activation may include molecules such as hypoxia inducible factor type 1 α (HIF-1α) and/or nitric oxide (NO). Using foam cells generated *in vitro*, Gessi et al. (2010) found that activation of A_3_AR, A_2B_AR, and to a lesser extent, the A_2A_ subtypes were associated with the production of VEGF induced by adenosine and hypoxia. This last effect was dependent on activation of ERK, p38 MAPK, Akt, and HIF-1α. Furthermore, adenosine has been reported to increase the synthesis of the angiogenic modulator NO in some, but not all, cultured endothelial cells (Sobrevia et al., 1996; Li et al., 1998; Wyatt et al., 2002). Whether, adenosine receptor-mediated activation of ERK-MAPK-HIF-1α increases NO is unknown. But, it has been reported that NO promotes a regulatory loop with ERK activation/deactivation (Schieke et al., 1999) and stabilization of HIF-1α and promotes HIF-1α binding to DNA (Kimura et al., 2000).

### EXPRESSION OF ADENOSINE RECEPTORS IN ENDOTHELIAL CELLS DURING HYPOXIA

The expression of adenosine receptor subtypes and their function are subject to dynamic regulation by hypoxia (Bshesh et al., 2002; Eltzschig et al., 2003; Feoktistov et al., 2004). Because the A_2B_AR promoter contains a functional binding site for HIF-1α (Kong et al., 2006), the onset of hypoxia strongly induces A_2B_AR expression in different cell types including human dermal microvascular endothelial cells (Eltzschig et al., 2003), and HUVEC (Feoktistov et al., 2004). In addition, elevated expression of A_2A_AR has also been reported after exposure to hypoxia in human placental homogenate (Von Versen-Hoynck et al., 2009), fetal chromaffin-derived cell line (Brown et al., 2011), and human lung endothelial cells, while no evidence of A_2A_AR upregulation was seen in mouse endothelial cells (Ahmad et al., 2009).

Interestingly, despite the fact that all adenosine receptors contain a hypoxia response element in their promoters (St. Hilaire et al., 2009), regulation via HIF is differentially modulated. Whereas A_2B_AR is regulated by HIF-1α, A_2A_AR is regulated by HIF-2α, suggesting that transcriptional regulation might be part of the switch of A_2A_AR toward A_2B_AR expression observed in HUVEC exposed to hypoxia (Feoktistov et al., 2004). This switch may have important functional implications for regulation of angiogenesis. For example, in HUVEC, adenosine does not stimulate VEGF secretion under normoxic conditions, but hypoxia increases the expression of A_2B_AR, which are then able to stimulate VEGF release (Feoktistov et al., 2004). Therefore, we could speculate that switching the expression of adenosine receptor toward A_2B_AR rather than A_2A_AR during hypoxia in the endothelium may offer some advantages in the angiogenic process, since high levels of adenosine may downregulate activation and/or expression of A_2A_AR as mechanisms of desensitization. But at the same time, upregulation of A_2B_AR will enhance or maintain the pro-angiogenic capacity of adenosine in conditions were high levels of this autocoid are expected. It is likely that this phenomenon may occur in preeclampsia.

## ADENOSINE, ANGIOGENESIS, AND PREECLAMPSIA

### ADENOSINE LEVELS DURING PREECLAMPSIA

The plasma level of adenosine is finely regulated by a series of enzymes responsible for synthesis and catabolism (see details in Escudero and Sobrevia, 2009). Compared with non-pregnant women, normal, pregnant women exhibit increased synthesis, but reduced catabolism, of adenosine (Yoneyama et al., 2000; Lee et al., 2007). Several studies have described high adenosine levels in both maternal (Yoneyama et al., 2001, 2002a,b,c) and fetal blood (Yoneyama et al., 1996; Espinoza et al., 2011; Escudero and Sobrevia, 2012) during preeclampsia, particularly in severe preeclampsia, compared with normal pregnancies (Yoneyama et al., 1996; Espinoza et al., 2011; Escudero and Sobrevia, 2012). Unexpectedly, these high levels are associated with high adenosine catabolism via adenosine deaminase 2 (ADA2; Yoneyama et al., 2002a; Kafkasli et al., 2006) as well as elevated adenosine uptake (Escudero et al., 2008). The causes and consequences of a high extracellular adenosine level in both maternal and fetal circulation during preeclampsia are unclear; however, it may be explained by an adaptive mechanism (Casanello et al., 2007; Escudero and Sobrevia, 2008, 2009, 2012; Escudero et al., 2009) associated with vasodilation or angiogenesis in preeclamptic placenta as occurs in other tissues, such as heart, muscle, or brain, in unfavorable conditions such as hypoxia (Eckle et al., 2007; Loffler et al., 2007).

The levels of adenosine in umbilical vein blood in preeclampsia (1.7 vs. 0.5 μmol/L, preeclampsia vs. normal pregnancy; Yoneyama et al., 1996; Espinoza et al., 2011) and in the culture medium of human placental microvascular endothelial cells (hPMEC) from preeclamptic pregnancies (2.7 vs. 0.6 μmol/L) are at least three times higher than in normal pregnancy (Escudero et al., 2008), making it likely that, in preeclampsia, all adenosine receptors are likely to be stimulated (Jacobson and Gao, 2006). However, only a few reports have described the effect of preeclampsia on the expression and function of adenosine receptors (Escudero et al., 2008, 2012; Kim et al., 2008; Von Versen-Hoynck et al., 2009). Thus, reduced expression of A_2A_AR, without changes in A_2B_AR (Escudero et al., 2008), was found in hPMEC isolated from preeclamptic placentas, whereas reduced A_2A_AR (Escudero et al., 2012) but higher A_2B_AR (Acurio et al., 2014) expression levels were found in HUVEC from preeclampsia. Yet, increased levels of all adenosine receptors have been reported in placental homogenate from preeclamptic placentas compared with normal pregnancy (Von Versen-Hoynck et al., 2009).

### ADENOSINE RECEPTOR ACTIVATION IN PREECLAMPSIA

It has been shown that activation of A_2A_AR leads to reduction in adenosine uptake by the equilibrative nucleotide transporter type 1 (hENT1) and hENT2, whereas A_2B_AR activation increases hENT2-mediated adenosine transport in cells from preeclamptic placentas (Escudero et al., 2008). Therefore, during preeclampsia, activation of adenosine receptors may control adenosine transport and, hence, extracellular adenosine levels. However, because adenosine levels are increased despite the elevation of total adenosine uptake, it is expected that the production of adenosine from sources such ATP or cell debris is higher in preeclampsia than in normal pregnancy (Spaans et al., 2014).

Recently, we observed reduced protein abundance of A_2A_AR in HUVEC derived from EOPE, but non-significant changes in LOPE, compared with cells from normal pregnancy. These findings were associated with a basal (i.e., without any treatment) reduction in cell migration/proliferation of HUVEC in EOPE compared with normal pregnancy or LOPE (Escudero et al., 2012). In addition, CGS-21680 (an A_2A_AR agonist) and NECA significantly increased HUVEC migration/proliferation in normal pregnancy, LOPE, and EOPE. However, considering that cells from EOPE exhibited the lowest migration/proliferation in the basal conditions, the magnitude of response to both adenosine receptor agonists in migration and proliferation tends to be higher in cells from EOPE than those from others groups. In agreement with these results, VEGF expression was significantly lower in HUVEC from EOPE, but higher in LOPE, compared to normal pregnancy. Also, CGS-21680 increases the protein abundance of VEGF in normal and EOPE-derived cells, an effect blocked by the A_2A_AR antagonist ZM-241385. Nevertheless, CGS-21680 did not affect VEGF expression in HUVEC from LOPE, but ZM-241385 led to a reduction (41 ± 6%, *p* < 0.05) in the level of this protein compared to corresponding levels at basal condition, suggesting that A_2A_AR is activated at basal condition in LOPE. Thus, A_2A_AR-mediated HUVEC proliferation and migration was associated with VEGF synthesis in normal pregnancy, LOPE, and EOPE.

To elucidate potential intracellular pathways related to A_2A_AR activation, we determined that CGS-21680 increased the synthesis of NO as evidenced by activation of eNOS (i.e., the p-eNOS/eNOS ratio) and nitrite and nitrotyrosine levels in HUVEC from normal pregnancies and EOPE, but not in LOPE. The stimulatory effect observed in normal and EOPE cells was blocked by ZM-241385 co-incubation. In contrast, ZM-241385 reduced NO synthesis in LOPE cells compared to non-treated controls. Furthermore, using the non-selective nitric oxide synthase inhibitor, L-NAME, we found a significant reduction in the HUVEC migration/proliferation responses and VEGF protein levels in cells from normal pregnancies and LOPE, but not in EOPE cells stimulated with CGS-21680.

**FIGURE 2:**
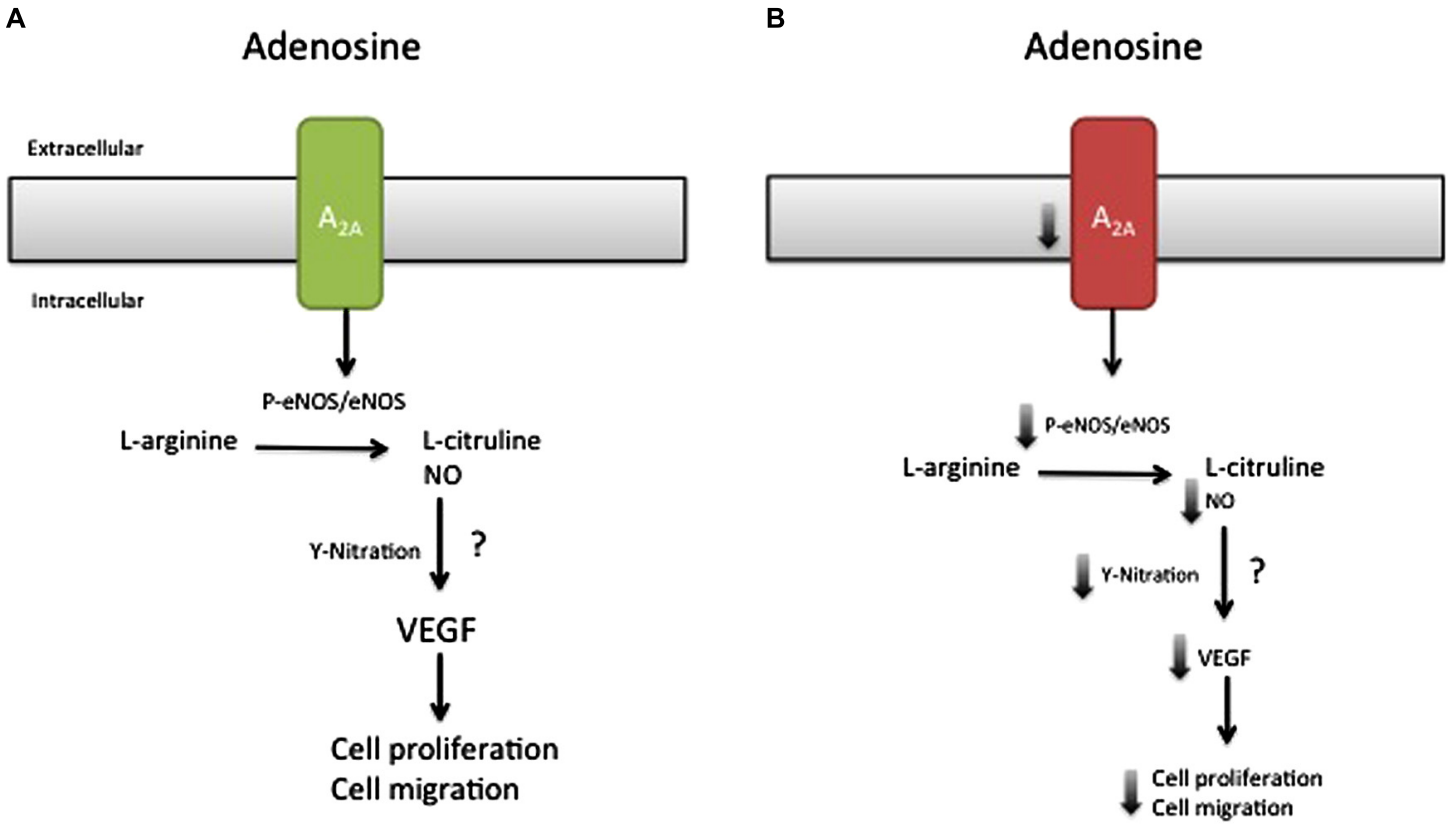
**Model of the effect of the A_2A_AR/NO/VEGF signaling pathway in cell migration and proliferation during preeclampsia.** Late-onset preeclampsia **(A)** is associated with elevated activation of A_2A_ adenosine receptor, characterized by high (↑) eNOS activation, NO formation, and nitrotyrosine formation (Y-nitration), associated with enhancement of cell proliferation and migration. On the other hand, early-onset preeclampsia **(B)** is associated with a reduction (↓) in the abundance of A_2A_ adenosine receptor, which in turn could be reduced with a decline in eNOS activation, NO formation, and Y-nitration and might explain the reduced HUVEC migration and proliferation observed in this disease. Despite this hypothesis, it is unclear (?) whether NO generated by A_2A_ stimulation can modulate the expression or activation of VEGF or activate other signaling pathways involved in cell migration and proliferation in early-onset preeclampsia.

Thus, our study demonstrated that activation of A_2A_AR is associated with the following cascade: eNOS activation (i.e., ser^1177^ phosphorylation), NO synthesis, nitrotyrosine formation, VEGF expression, and cell proliferation/migration in normal pregnancy. However, cells derived from EOPE and LOPE were different in several aspects. Whereas EOPE cells exhibited low A_2A_AR expression and reduction of NO/VEGF synthesis and cell proliferation/migration; LOPE cells demonstrated increased cell proliferation/migration, mediated in part through the same pathway (see **Figure 2**). Existence of this pathway was recently confirmed using selective shRNA for A_2A_AR in HUVEC. Knockdown of A_2A_AR was associated with reduced formation of intracellular cAMP, NO metabolites, VEGF protein level, and the capacity for tube formation compared with controls (unpublished results).

As stated before, adenosine-dependent angiogenesis can be regulated by all four adenosine receptors. There is little information on the role of A_2B_AR in the angiogenic process during preeclampsia, whereas the participation of A_1_AR and A_3_AR in this process is unknown. In primary cultures of hPMEC, a cell type with high pro-angiogenic capacity compared to HUVEC (Dye et al., 2004), we found that A_2B_AR may be constitutively activated in cells from preeclamptic placentas, since the use of A_2A_/A_2B_AR inhibitors in non-stimulated cells decreases adenosine uptake (Escudero et al., 2008). More recently, we found that activation of A_2B_AR in HUVEC accounts for at least 30% of the pro-proliferative response mediated by adenosine or NECA (Acurio et al., 2014). These data agree with prior reports (Feoktistov et al., 2002, 2004) that exposure of HUVEC to hypoxia increases the expression of A_2B_AR, which is then able to stimulate VEGF release.

### HYPOTHESIS FOR A ROLE FOR ADENOSINE IN PREECLAMPSIA

In view of available information, we can speculate that during preeclampsia, a condition associated with reduction in the expression and activity of A_2A_AR, a compensatory increase in the expression and/or activity of A_2B_AR occurs that tends to compensate the impaired adenosine-mediated pro-angiogenic process. Moreover, since adenosine is pro-angiogenic, the reduction in A_2A_AR expression and down activation of A_2A_AR-dependent intracellular pathway might be part of the apparent “adenosine paradox,” in which increased adenosine levels do not stimulate angiogenesis in preeclampsia. The mechanism underlying this phenomenon is unclear, but may be associated with the capacity of adenosine to regulate the expression of its receptors, as exhibited in cells such as cardiomyocytes (Headrick et al., 2013) or PC12 cells (Saitoh et al., 1994). In particular, PC12 exposure to A_2A_AR agonists reduces ADORA2 gene expression (Saitoh et al., 1994), suggesting a transcriptional regulation of A_2A_AR by adenosine. Whether similar regulation is present in endothelium is unknown, but this is a possible mechanism for the pathophysiological deregulation observed in preeclampsia.

**FIGURE 3:**
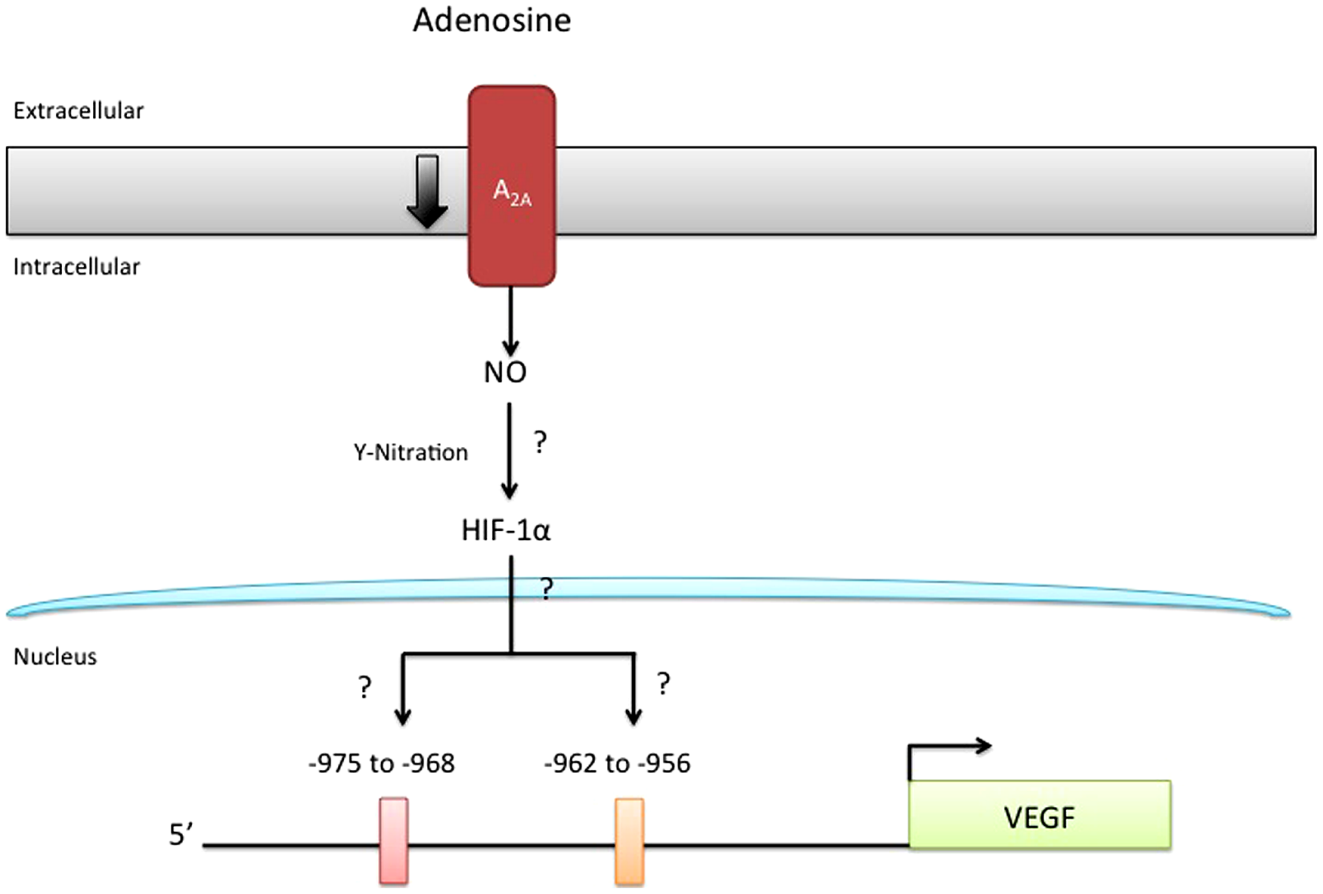
**Reduced A_2A_AR/NO signaling pathway may involve HIF nitration.** It has been hypothesized that nitration of hypoxia inducible factor (HIF) may control the expression of several genes, including vascular endothelial growth factor (VEGF). Since adenosine requires HIF for the induction of VEGF-promoter activity and because this receptor triggers an intracellular signaling involving protein nitration in normal and preeclamptic endothelial cells, it is unknown (?) whether HIF-nitration after A_2A_AR stimulation might control HIF-binding to a specific site (including –975 to –968; and –962 to –956) in the VEGF promoter.

Although the intracellular signaling pathway related to adenosine receptor activation is an area of active research, only our recent study suggested a potential adenosine receptor-dependent mechanism in preeclampsia. On the basis of this study, we propose a model (**Figure 2**), in which low expression of A_2A_AR in EOPE leads to reduction in NO and VEGF expression (Escudero et al., 2012). The implication of these alterations for feto-placental angiogenesis is poorly understood, but might involve changes in the activation of HIF (Kimura et al., 2002) and changes in the promoter activity of several proteins, including anti-angiogenic factors such as thrombospondin 2 (MacLauchlan et al., 2011) or pro-angiogenic factors like VEGF (Kimura et al., 2002; Feoktistov et al., 2004). Considering that NO can cause nitration of tyrosine residues on HIF-1α (Riano et al., 2011), and may contribute to stabilization, we propose that the reduced adenosine-mediated NO synthesis observed in EOPE might be associated with impaired HIF-dependent VEGF expression (see **Figure 3**). Clearly, more studies are necessary to understand all the processes involved in these alterations.

Another question that needs to be answered is whether impaired adenosine-mediated angiogenesis in the feto-placental circulation of preeclamptic pregnancies persists after birth. In this context, Coney and Marshall (2010) have demonstrated that prenatal hypoxia has long-lasting effects on vascular function in the skeletal muscle of adult male rats. In particular, in a group of adult males, they investigated how chronic systemic hypoxia *in utero* (CHU) affects the cardiovascular response evoked by acute, systemic hypoxia. One of the most intriguing results was the fact that the overall magnitude of vasodilator response evoked in muscle by acute systemic hypoxia is similar in CHU and normoxic rats, but the mechanisms underlying the response appear to be different. Thus, they conclude that, whereas in normoxia, vasodilatory response is associated with the activation of endothelial A_1_AR and NO-dependent effects, in CHU, participation of A_1_AR is limited, and vasodilatory response in the muscle is replaced by factors other than adenosine. Moreover, it has been reported that mice deficient in A_2A_AR (KO-A_2A_AR) exhibit no significant difference in systemic blood pressure compared to wild-type animals, but they do develop pulmonary artery hypertension and pulmonary vascular remodeling (Xu et al., 2011). Thus, these studies demonstrate how the adenosine-impaired angiogenesis and vascular remodeling observed in pathological pregnancies such as preeclampsia may be related to future cardiovascular risk.

## CONCLUDING REMARKS AND FUTURE DIRECTIONS

That fetuses exposed to preeclampsia are at increased risk to develop hypertension later in life has been associated with the occurrence of endothelial dysfunction. Since the endothelium is one of the main factors in the normal process of angiogenesis, an impaired endothelial/angiogenic response in offspring from preeclamptic pregnancies may constitute the underlying mechanism associated with hypertension. On the other hand, preeclampsia is associated with elevated levels of adenosine and low expression and response of A_2A_AR, but high expression of A_2B_AR, defects that might be involved in abnormal placental and newborn angiogenic processes. Then, adenosine may constitute a potential new target for improving placental angiogenesis. Furthermore, impairment of those mechanisms may contribute to susceptibility to cardiovascular diseases, including hypertension, in children exposed to preeclampsia.

As presented in this review, there are many questions that need to be answered regarding adenosine-mediated angiogenesis in preeclampsia. Therefore, future studies should consider at least the following inquires. Why A_2A_AR expression is reduced in preeclampsia? Future studies should consider analysis of translational and transcriptional regulation of A_2A_AR expression in endothelial cells derived from preeclampsia. Also, it should be determined whether the reduction in total A_2A_AR levels observed in preeclampsia, leads to a reduction in the cell surface expression and in the activation of this receptor. Studies are also needed to investigate how cross-talk between intracellular pathways related to adenosine receptor activation might change during preeclampsia; and how these phenomena might generate a compensatory response via other adenosine receptors including A_2B_AR. As highlighted in this review, it is also necessary to determine whether adenosine-mediated angiogenesis is present after birth in newborns and children exposed to preeclampsia. These studies are difficult to perform in humans due to ethical and technical issues; but certainly animal models might help. Mice deficient in each one of the adenosine receptors have been developed, providing an excellent model to address these last questions. We hope that this review will contribute to awareness, within the scientific community, of this important issue and stimulate further investigation in this area.

## Conflict of Interest Statement

The authors declare that the research was conducted in the absence of any commercial or financial relationships that could be construed as a potential conflict of interest.

## ABBREVIATIONS

NECA: 5′-*N*-ethylcarboxamido-adenosine
AP-1: Activator protein 1
AR: adenosine receptor
A_2A_AR: adenosine receptor A_2A_
A_2B_AR: adenosine receptor A_2B_
bFGF: basic fibroblast growth factor
EOPE: early-onset preeclampsia
eNOS: endothelial nitric oxide synthase
EPC: endothelial progenitor cells
hENT1: equilibrative nucleotide transporter type 1
hENT2: equilibrative nucleotide transporter type 2
Epac: exchange protein activated by cAMP
ERK: extracellular signal-regulated kinase
hPMEC: human placental microvascular endothelial cell
HUVEC: human umbilical vein endothelial cell
HIF-1α: hypoxia inducible factor type 1 α
IL-8: interleukin 8
LOPE: late-onset preeclampsia
MAPK: mitogen-activated protein kinase
LLC: mouse Lewis lung carcinoma
NO: nitric oxide
PKA: protein kinase A
sFlt-1: soluble vascular endothelial growth factor receptor type 1 or soluble fms-like tyrosine kinase-1
sEnd: soluble endoglin
VEGF: vascular endothelial growth factor.
